# Measurement of metabolite variations and analysis of related gene expression in Chinese liquorice (*Glycyrrhiza uralensis*) plants under UV-B irradiation

**DOI:** 10.1038/s41598-018-24284-4

**Published:** 2018-04-18

**Authors:** Xiao Zhang, Xiaoli Ding, Yaxi Ji, Shouchuang Wang, Yingying Chen, Jie Luo, Yingbai Shen, Li Peng

**Affiliations:** 1 College of Biological Sciences and Technology, Beijing Forestry University, Beijing, 100083 China; 2 National Engineering Laboratory for Tree Breeding, Beijing Forestry University, Beijing, 100083 China; 3 Key Lab of Ministry of Education for Protection and Utilization of Special Biological Resources in Western China, Ningxia University, Yinchuan, Ningxia 750021 China; 4 School of Life Science, Ningxia University, Yinchuan, Ningxia 750021 China; 5 National Key Laboratory of Crop Genetic Improvement and National Center of Plant Gene Research (Wuhan), Huazhong Agricultural University, Wuhan, Hubei 430070 China

## Abstract

Plants respond to UV-B irradiation (280–315 nm wavelength) via elaborate metabolic regulatory mechanisms that help them adapt to this stress. To investigate the metabolic response of the medicinal herb Chinese liquorice (*Glycyrrhiza uralensis*) to UV-B irradiation, we performed liquid chromatography tandem mass spectrometry (LC-MS/MS)-based metabolomic analysis, combined with analysis of differentially expressed genes in the leaves of plants exposed to UV-B irradiation at various time points. Fifty-four metabolites, primarily amino acids and flavonoids, exhibited changes in levels after the UV-B treatment. The amino acid metabolism was altered by UV-B irradiation: the Asp family pathway was activated and closely correlated to Glu. Some amino acids appeared to be converted into antioxidants such as γ-aminobutyric acid and glutathione. Hierarchical clustering analysis revealed that various flavonoids with characteristic groups were induced by UV-B. In particular, the levels of some ortho-dihydroxylated B-ring flavonoids, which might function as scavengers of reactive oxygen species, increased in response to UV-B treatment. In general, unigenes encoding key enzymes involved in amino acid metabolism and flavonoid biosynthesis were upregulated by UV-B irradiation. These findings lay the foundation for further analysis of the mechanism underlying the response of *G*. *uralensis* to UV-B irradiation.

## Introduction

Plants inevitably encounter environmental stresses due to their sessile nature. With the depletion of the stratospheric ozone layer, an increasing amount of UV-B radiation has been reaching the Earth’s surface and the surfaces of plants^1^. Although it constitutes a very small fraction of the solar spectrum, UV-B radiation (280–315 nm) is a potent environmental factor that influences many aspects of a plant’s life, including growth, development, and morphology^2,3^. Plants have evolved ways to alter their metabolism and reconfigure metabolic networks in response to UV-B stress. Plants produce elevated levels of several amino acids (i.e., Ala, Glu, Lys, and Phe) and some of their derivatives (i.e., γ-amino butyrate) under UV-B irradiation^4^. This response occurs at the primary metabolic level after short-term UV-B treatment, which then triggers elaborate changes in the production of functional secondary metabolites that can protect against UV-B damage^4,5^. Secondary metabolites accumulate in plants under UV-B irradiation^6–10^. In particular, UV-B irradiation alters the phenylpropanoid pathway, triggering a marked accumulation of flavonoids^4,11^, which may help alleviate the damaging effects of irradiation by absorbing various UV wavelengths^12–14^. However, flavonoids are less effective at UV-B absorption than hydroxycinnamic acid derivatives^6^, which might function in UV screening, whereas flavonoids might primarily serve as antioxidants during photoprotection^15–17^. UV-B also is a general source of oxidative stress^11,18,19^ and triggers the production of free radical scavengers of enzymatic or non-enzymatic systems in plants in response to oxidative damage^2,20^. In *Catharanthus roseus*, the increased contents of glutathione (GSH) and several alkaloids under UV-B irradiation, as well as the increased activities of the enzymes superoxide dismutase (SOD) and peroxidase (POD), can be attributed to the generation of reactive oxygen species (ROS) induced by UV-B^21^.

Chinese liquorice (*Glycyrrhiza uralensis*), one of the oldest Chinese medicinal herbs, has high levels of bioactive phytochemicals^22,23^. This plant has multiple pharmacological properties, including anti-inflammatory, antiviral, antimicrobial, antioxidative, anticancer, and antidiabetic effects^24–27^. *G*. *uralensis* is mainly found in arid, semi-arid, and desert areas of northwest China and is therefore exposed to harsh conditions such as drought, cold, high-salinity soil, and UV-B radiation^28,29^. These stresses may induce the activity of various metabolic pathways that contribute to the stress resistance of *G*. *uralensis*. Drought and UV-B irradiation promote the accumulation of secondary metabolites in the roots of *G*. *uralensis*^30–32^. However, little is known about the metabolic and transcriptomic changes that occur in *G*. *uralensis* leaves in response to UV-B irradiation. Leaves are the direct recipients of UV-B irradiation and they play a vital role in carbohydrate, amino acid, and secondary metabolite biosynthesis in plants due to their primary function in photosynthesis. Hence, the quality of light absorbed by leaves affects plant growth and development through regulating the biosynthesis and breakdown of various metabolites^33,34^.

In the present study, we performed liquid chromatography tandem mass spectrometry (LC-MS/MS) to investigate the metabolites variations of *G*. *uralensis* leaves in response to UV-B irradiation. We also performed transcriptome analysis to explore the mechanism underlying the metabolic variation and regulation in *G*. *uralensis* leaves induced by UV-B irradiation, shedding light on the stress response in this important medicinal herb.

## Results

### Non-targeted metabolomics analysis of *G*. *uralensis* leaves under different periods of UV-B irradiation treatment

**Figure 1:**
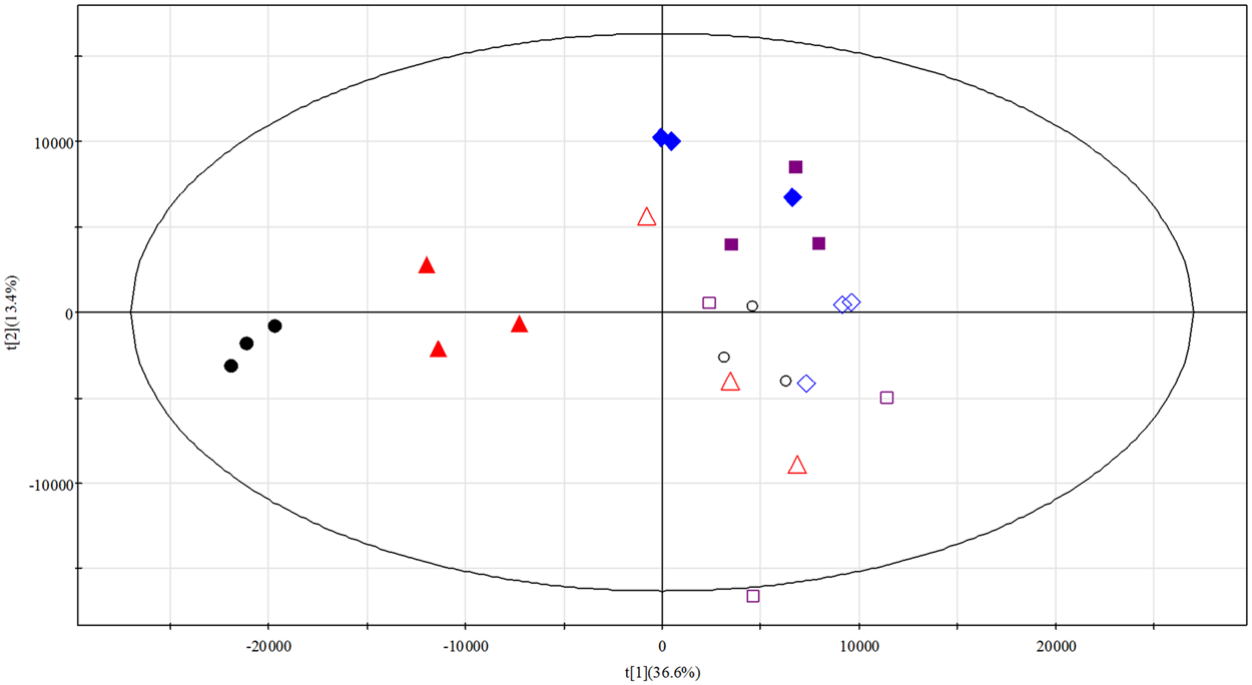
PCA score plot of metabolites in *Glycyrrhiza uralensis* leaves from control seedlings and UV-B irradiated seedlings at four time points. Solid squares, diamonds, triangles, and dots represent leaf samples from seedlings irradiated by UV-B for 12, 24, 48, and 96 h, respectively. Open squares, diamonds, triangles, and circles represent leaf samples from control seedlings at 12, 24, 48, and 96 h, respectively.

To investigate the effects of different periods of UV-B irradiation on the metabolism of *G*. *uralensis* leaves, we exposed the plants to UV-B radiation for four time points. We also collected four control samples of plants exposed to regular white light at the same time points (Supplementary Fig. S1). Leaf samples were subjected to liquid chromatography-time-of-flight-mass spectrometry (LC-TOF-MS) and based on non-targeted metabolomics, principal component analysis (PCA) was performed to evaluate the variations in the levels of metabolites. The results found that the first two principal components explained 36.6% and 13.4% of the metabolic variances for all samples (Fig. 1), and loadings of the two PCs (PC1 and PC2) are listed in Supplementary Table S1. According to the PCA score plot, the four groups of controls were located close together and could not be separated, indicating that the metabolic components of the four control groups of seedlings were similar. Samples that were UV-B irradiated for more than 48 h were separated by PC1 from their respective controls, and this separation became more severe with the increasing time of UV-B irradiation. These results indicated that UV-B did not cause metabolic changes after 12 and 24 h of irradiation, but it induced marked metabolic differences in leaves after more than 48 h irradiation, with the most prominent metabolic profile changes observed in seedlings treated with 96 h UV-B irradiation, compared to the controls. Therefore, the PCA score plot indicates that the UV-B radiation treatment induced metabolic variations of *G*. *uralensis* leaves, especially after 48 and 96 h of treatment.

### Metabolite identification/annotation in *G*. *uralensis* leaves

To explore the response to UV-B in *G*. *uralensis* leaves, we examined the metabolites in leaves subjected to irradiation and the controls. Based on non-targeted metabolic profiling analysis by an HPLC-ESI-QTOF-MS/MS system^35^, we acquired almost 1000 signals (Supplementary Table S1). Precursor ions which were ranked higher by OPLS-DA VIP score (VIP > 1) (Supplementary Table S2) were subjected to the targeted MS^2^ mode to obtain fragmentation patterns, and subsequently a MS/MS spectral tag (MS2T) library containing 170 high-quality (*s*/*n* > 10), highly reproducible and (almost) non-redundant metabolite signals with the product ion spectra (MS^2^) was created (Supplementary Table S3). The database was then used to identify/annotate metabolites based on the accurate *m*/*z* values, retention time (RT), and fragmentation patterns. In terms of the annotation, metabolites with available commercial standards were identified by comparing the *m*/*z* values, RT, and fragmentation patterns of metabolites with those of the standards, which were analyzed by employing the same profiling procedure used for the plant extracts. For the metabolites with no available standards, their fragmentation patterns were compared with what have been published in literatures or databases (MassBank, KNApSAcK and METLIN), best matches were then searched in KEGG for possible structures.

**Figure 2:**
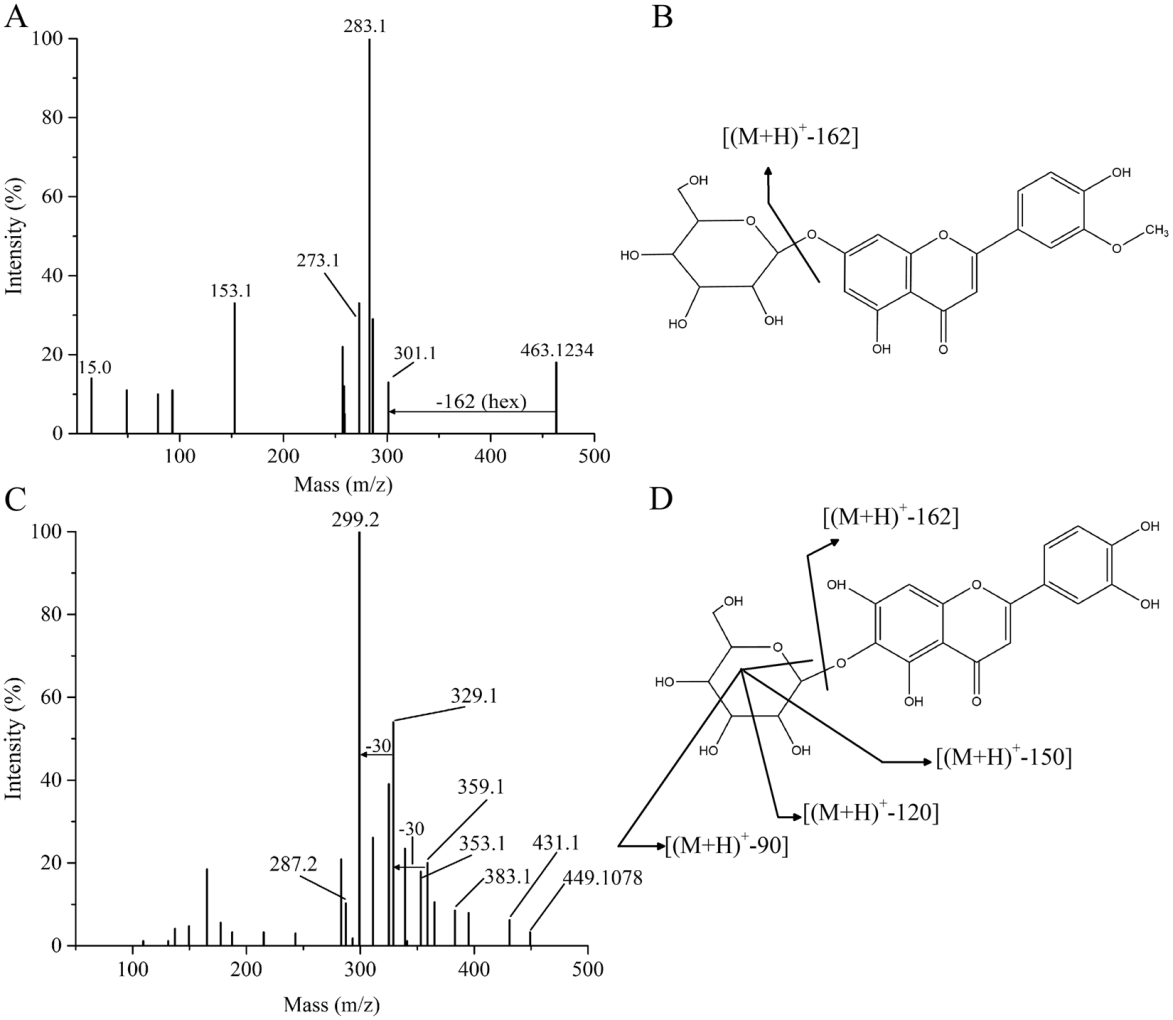
Identification of flavonoids in *Glycyrrhiza uralensis* leaves. (**A**) MS/MS spectra of m/z 463.1234 and the metabolite was putatively identified as Chrysoeriol 7-*O*-hexoside. (**B**) Structure and fragmentation of Chrysoeriol 7-*O*-hexoside. (**C**) MS/MS spectra of m/z 449.1078 and the metabolite was identified as Luteolin 6-*C*-glucoside with authentic standard. (**D**) Structure and fragmentation of Luteolin 6-*C*-glucoside.

We putatively identified more than 54 metabolites by the procedures mentioned above (Table 1), and structure elucidations of four metabolites each representing one type of flavonoids were described as examples, including *O*-glycosylflavonoid, *C*-glycosylflavonoid, di-*C*, *C*-glycosylflavonoid and *C*, *O*-glycosylflavonoid. For instance, compound m162 (RT 8.99 min) was putatively identified as chrysoeriol 7-*O*-hexoside with a [M + H]^+^ ion at *m*/*z* 463.1234 in positive ionization mode, and it fragmented at *m*/*z* 301 which indicated its aglycone ion (chrysoeriol) and the loss of a hexose (162 amu) (Fig. 2A). Compound m051 (RT 7.16 min) was identified as luteolin 6-*C*-glucoside with a [M + H]^+^ ion at 449 and its aglycone ion (luteolin) at 287, typical ions of the *C*-glycosylated flavones fragmentation were observed^36,37^: 431 [(M + H)−18]^+^, 359 [(M + H) − 90]^+^, 329 [(M + H) − 120]^+^, and 299 [(M + H) − 150]^+^ (Fig. 2C). Structures and main fragmentation of this two compound are presented in Fig. 2B and D. Compound m170 (RT 7.02) was putatively identified as *C*-pentosyl-*C*-hexosyl-apigenin with a [M + H]^+^ ion at *m/z* 565.1553 in positive ionization mode. It fragmented at *m/z* 294 due to the loss of an apigenin moiety (271 amu). It presented several fragment ions: *m/z* 505 [(M + H) − 60]^+^, *m/z* 475 [(M + H) − 90]^+^, *m/z* 469 [(M + H) − 96]^+^, *m/z* 445 [(M + H) − 120]^+^, *m/z* 415 [(M + H) − 150]^+^, *m/z* 325 [(M + H) − 150–90]^+^, *m/z* 295 [(M + H) − 150 − 120]^+^. This fragmentation pattern possibly indicated the simultaneous fragmentation of two sugar moieties, with typically losses of 90, 96, 120 and 150 amu were for hexoses, and 60, 90 and 120 amu for pentoses, respectively^38^ (Supplementary Fig. S2A,B). An aromatic acylated *C*-*O*-glycosylflavonoid (compound m096, *m/z* 801.2243, RT 8.19 min) was putatively identified as *C*-hexosyl-chrysoeriol *O*-feruloylhexoside: *m/z* 463, 397, 367, 343 and 313 were the diagnostic fragment ions of *C*- hexosyl-chrysoeriol, and *m/z* 177 was a diagnostic fragment ion of ferulic acid^38,39^. Structures and main fragmentation of this compound are presented in Supplementary Fig. S2C,D.

**Table 1:** A list of 54 metabolites identified/annotated in *Glycyrrhiza uralensis* leaves.

Metabolite names	RT (min)	m/z	Main fragments
Alanine	1.57	90.9750	44.0
Arginine	1.41	175.1991	116.1, 157.2, 130.0, 65.4
Asparagine	1.72	133.0450	74.0, 123.1, 112.1, 83.1
Aspartic acid	1.57	134.0986	88.1, 116.1, 74.1, 70.0, 68.0
Glutamic acid	1.57	148.0533	84.1, 130.1, 102.1, 79.7
Histidine	1.42	156.0859	110.1, 93.1, 83.1, 67.4
Isoleucine	1.72	132.1990	86.2, 69.1, 67.1, 56.1
Leucine	2.44	132.0986	86.1, 69.1, 67.1, 56.1
Lysine	1.57	147.1878	84.2, 130.1
Methionine	1.64	150.2420	61.2, 104.3, 75.0, 81.0, 53.9
Phenylalanine	3.67	166.1763	120.1, 103.7, 93.4, 72.2
Proline	1.57	116.0633	70.0, 66.6, 63.9
Serine	1.78	106.0925	60.0
Threonine	1.57	120.0637	74.0, 55.8
Tryptophan	4.95	205.1994	146.2, 118.1, 170.3, 188.2, 91.0
Tyrosine	2.41	182.2013	91.2, 136.2, 119.3, 76.9, 136.2
Valine	1.64	118.0789	72.2, 69.9, 52.4
Glutathione (oxidized)^N^	2.40	612.1558	355.7, 484.3, 248.5, 305.2, 538.5, 191.0
Apigenin	11.67	271.0601	153.1, 243.2, 229.1, 197.2, 145.2
*C*-hexosyl-apigenin *O*-feruloylhexoside^N^	8.24	771.2132	177.1, 433.1, 337.0, 591.1, 145.1
*C*-hexosyl-apigenin *O*-*p*-coumaroylhexoside^N^	8.60	741.2027	147.2, 579.3, 367.1, 337.0, 207.2
*C*-hexosyl-chrysoeriol *O*-feruloylhexoside^N^	8.19	801.2243	463.1, 397.1, 367.1, 177.1, 343.1, 313.1
*C*-hexosyl-luteolin *O*-feruloylhexoside^N^	7.58	787.2082	449.1, 383.1, 329.1, 177.1
Chrysoeriol	11.93	301.0707	286.0, 258.1
Chrysoeriol 5-*O*-hexoside^N^	8.39	463.1233	301.1, 286.3, 258.4
Chrysoeriol 7-*O*-hexoside^N^	8.99	463.1234	301.1, 286.3, 258.4
Chrysoeriol *C*-hexoside^N^	8.09	463.1239	313.1, 367.0, 343.0, 298.0
*C*-pentosyl-apeignin *O*-feruloylhexoside^N^	9.23	741.2027	177.1, 145.1, 283.3, 367.2
*C*-pentosyl-apigenin *O*-hexoside^N^	8.32	565.1554	367.1, 403.0, 337.1, 313.1, 283.0
*C*-pentosyl-apigenin *O*-*p*-coumaroylhexoside^N^	9.20	711.1919	367.1, 549.4, 349.1, 337.1, 283.2
*C*-pentosyl-*C*-hexosyl-apigenin^N^	7.02	565.1553	505.2, 547.3, 475.2, 445.2, 511.3, 415.2
*C*-pentosyl-chrysoeriol *O*-feruloylhexoside^N^	9.18	771.2120	177.0, 433.1, 337.0, 591.1, 145.1
*C*-pentosyl-chrysoeriol *O*-hexoside^N^	8.50	595.1663	397.1, 433.1, 379.2, 367.2, 343.2
*C*-pentosyl-luteolin *O*-hexoside^N^	7.70	581.1505	383.2, 419.1, 365.2, 353.2, 329.2
di-*C*,*C*-hexosyl-methylluteolin^N^	5.99	625.1766	607.2, 463.2, 391.1, 367.1, 343.1
di-*C*,*C*-pentosyl-apigenin^N^	7.62	535.1438	499.3, 481.3, 433.4, 381.5, 349.7
di-*C*,*C*-pentosyl-luteolin^N^	7.21	551.1398	497.1, 515.2, 407.1, 395.1, 365.1
Eriodictyol *C*-hexoside^N^	7.29	450.9858	331.1, 433.1, 385.1, 355.1, 301.2
Eriodictyol *O*-malonylhexoside^N^	9.90	536.9119	289.0
Luteolin	10.6	287.0544	153.0, 135.0
Luteolin 6-*C*-glucoside	7.16	449.1078	299.2, 359.1, 329.1, 283.2
Luteolin *C*-hexoside isomer ^N^	7.50	449.1087	299.2, 413.1, 353.1, 329.2
Luteolin *O*-malonylhexoside^N^	10.2	534.9318	287.0
Naringenin	11.7	273.0757	153.0, 147.0, 119.0
Naringenin *O*-malonylhexoside^N^	10.7	520.9838	273.0
Quercetin *O*-malonylhexoside^N^	9.90	551.0106	303.0
Resokaempferol 7-*O*-hexoside^N^	9.20	433.1137	271.1, 379.2, 313.1, 153.2
Sakuranetin	14.44	286.9843	167.1, 153.2
Tricetin *O*-malonylhexoside^N^	10.80	550.9927	303.0
Tricin	11.85	331.0811	315.0, 285.0, 258.0, 243.0, 153.0
Tricin 5-*O*-hexoside derivative^N^	9.42	535.1091	331.1, 315.0, 287.1, 270.1, 233.2
Tricin derivative^N^	9.52	647.2059	331.0, 315.0, 270.1, 157.2
Tricin *O*-hexoside derivative^N^	13.26	671.1965	509.1, 631.4, 491.1, 331.1
Tricin *O*-malonylhexoside^N^	10.52	579.1344	331.1, 535.1, 493.1, 315.2, 270.2

*Indicate no standard compound provided.

Based on the annotation, 24 metabolites were identified after comparing with authentic standards. Multiple reaction monitoring (MRM) was subsequently performed by LC-ESI-Q TRAP-MS/MS for quantification of 54 identified/putatively identified metabolites (Supplementary Table S4).

### Variations in identified/annotated metabolites over time in *G*. *uralensis* leaves under UV-B radiation

**Figure 3:**
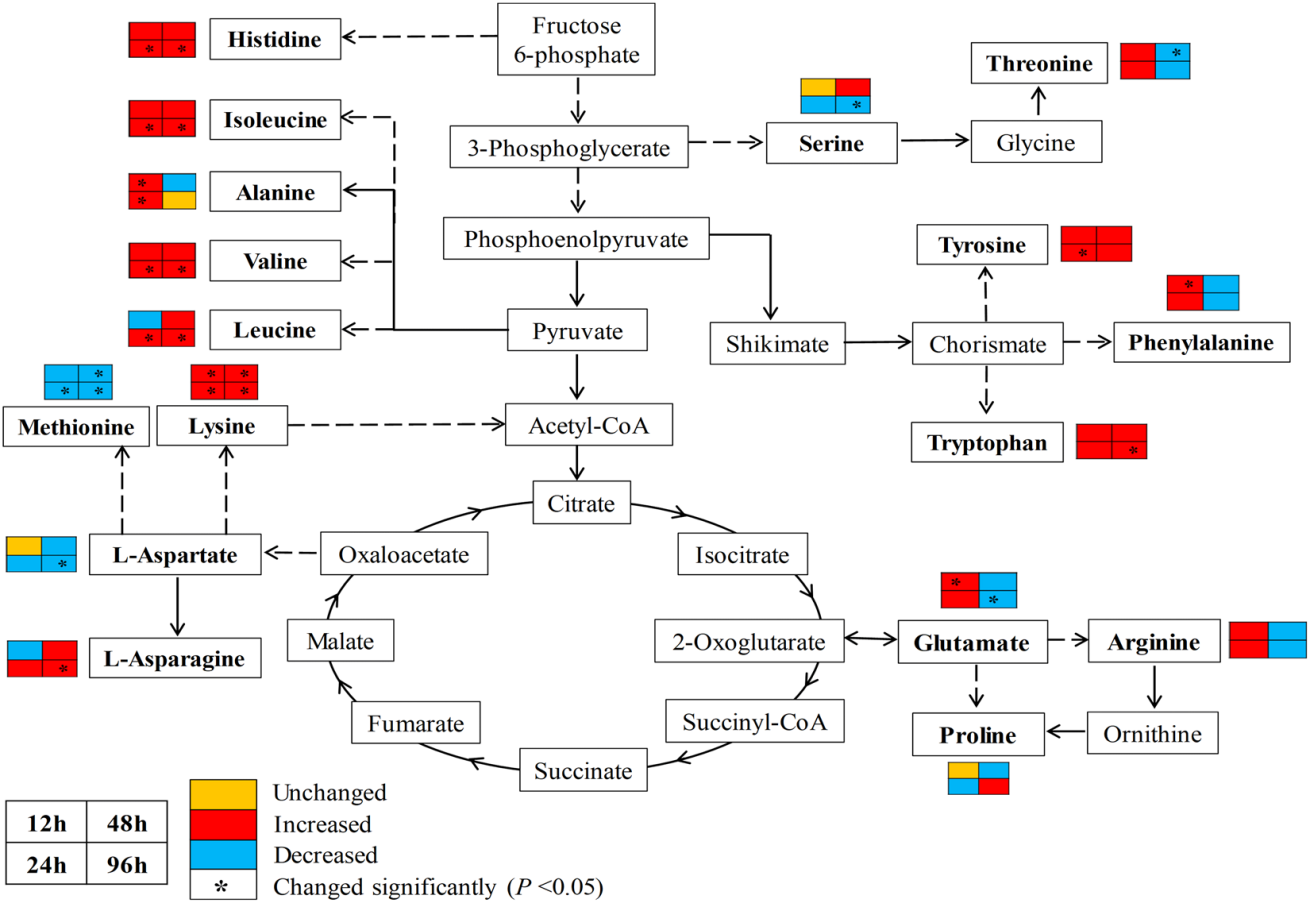
Changes in amino acid levels in the context of amino acid biosynthesis pathways in *Glycyrrhiza uralensis* leaves under UV-B irradiation at four time points. Metabolites detected shown in bold, solid lines represent one-step reactions, and dashed lines represent multi-step reactions. The boxes around metabolites indicate the ratios of amino acid contents in leaves under UV-B irradiation compared to the controls. Yellow, red, and blue indicate unchanged, increased, and decreased levels, respectively. Values of contents are means of 3 biological replicates with independent plants. The small boxes in the left top, left bottom, right top, and right bottom sides of each colored box indicate altered levels at 12, 24, 48, and 96 h, respectively. Significantly altered expression (*P* < 0.05) is indicated by an asterisk in the boxes.

In this work, we examined the variation of the identified/annotated metabolites over time, including amino acids and flavonoids. Analysis of the normalized fold changes in amino acid contents performed by hierarchical cluster analysis (HCA) showed that among the 17 amino acids detected in *G*. *uralensis* leaves, the levels of two aliphatic amino acids (Val and Ile), two aromatic amino acids (Tyr and Trp), and two basic amino acids (His and Lys) increased in seedlings under various periods of UV-B irradiation compared to the controls (Supplementary Fig. S3). By contrast, Asp and Met levels decreased at all four time points compared to the control (Supplementary Fig. S3). The levels of some amino acids changed at specific time points after UV-B radiation, such as Phe, Thr, and Glu. The contents of these amino acids increased at 12 and 24 h of UV-B irradiation but decreased at 48 and 96 h (Supplementary Fig. S3). These results suggest that short-term UV-B irradiation might activate the biosynthesis of Phe, Thr, and Glu in *G*. *uralensis* seedlings, whereas these amino acids might be degraded or used to synthesize other metabolites in response to UV-B radiation after more than 24 h of treatment. To better understand the variation in individual amino acids within the metabolic network of *G*. *uralensis* leaves in response to different lengths of UV-B radiation, we allocated the amino acids identified in our study to common amino acid biosynthetic pathways. A diagram of the changes in amino acid contents in primary metabolic pathways in *G*. *uralensis* under UV-B irradiation at all four time points compared to the control is shown in Fig. 3; amino acids with significantly altered levels are indicated in the diagram (*P* < 0.05, Supplementary Table S5).

**Figure 4:**
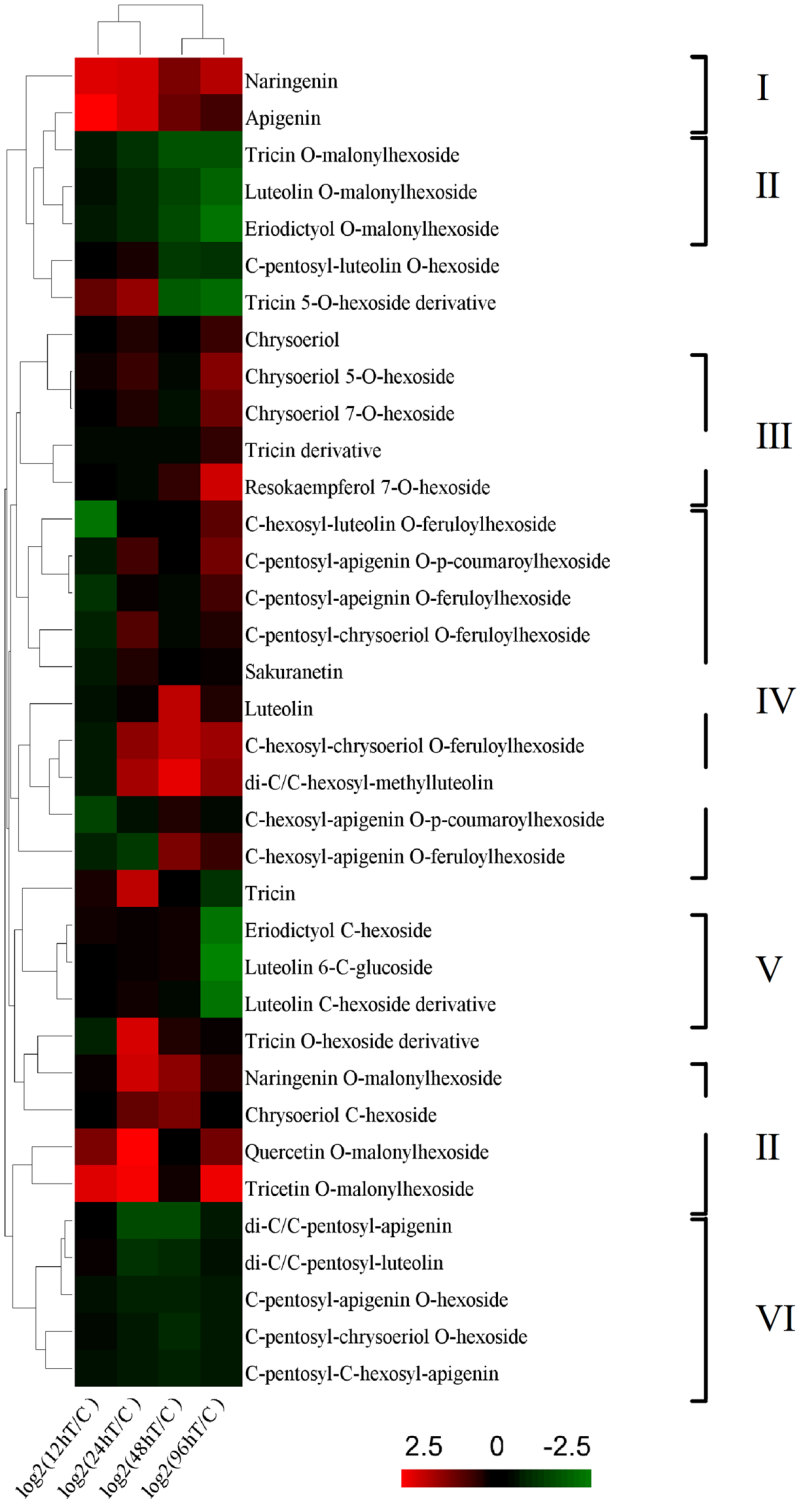
Variations in flavonoid levels in *Glycyrrhiza uralensis* leaves under UV-B irradiation compared to the controls at four time points. The fold change value of each treatment compared to the control was normalized to complete hierarchical clustering analysis. Each flavonoid is represented in a single row, and log_2_ (fold change) of each time point is represented in a single column. Red indicates higher flavonoid contents in treated seedlings than the control, whereas green indicates lower flavonoid contents in treated seedlings than the controls (a key is shown to the bottom of the heat map).

To further investigate the variation in flavonoid levels in *G*. *uralensis* leaves under different periods of UV-B irradiation, we normalized the fold changes (UV treated/controls) of various flavonoids and subjected them to hierarchical cluster analysis. Flavonoids with distinct modification groups exhibited diverse variation patterns during the time course of UV-B irradiation (Fig. 4). The contents of flavonoids without any glycosylation, including apigenin and naringenin (Fig. 4-I), were higher in UV-B-treated leaves compared to the controls. The levels of apigenin in leaves treated with 12 h irradiation were ten-fold more than those of the controls (Supplementary Table S5), suggesting that increased amounts of apigenin are synthesized within a short period in *G*. *uralensis* under UV-B radiation. Flavonoids with glycosylation detected in our results can be divided into several kinds according to their modification groups, including *C*/*O* monoglycosylation, di-glycosylated flavonoids with aromatic acylated and di-glycosylated flavonoids without any acylation. For malonylated flavonoids with *O*-monoglycosylation (Fig. 4-II), their levels exhibited two kinds of changes. In the first group, comprising the *O*-malonylhexosides of tricetin, quercetin, and naringenin, the levels increased after UV-B irradiation. In the second group, the levels of other *O*-malonylhexosides, including tricin *O*-malonylhexoside, eriodictyol *O*-malonylhexoside, and luteolin *O*-malonylhexoside, decreased to some degree during the course of UV-B irradiation. And for flavonoids of *O*-monoglycosylation without malonylation (Fig. 4-III), including chrysoeriol 5-*O*-hexoside, chrysoeriol 7-*O*-hexoside and resokaempferol 7-*O*-hexoside, their contents changed not much except that increased levels were observed under treatment of 96 h. For flavonoids with *C*-monoglycosylation (Fig. 4-V), such as eriodictyol *C*-hexoside and luteolin 6-*C*-glucoside, the levels did not change much after UV-B irradiation for 12, 24, and 48 h, but decreased after 96 h of treatment. For aromatic acylated di-glycosylated flavonoids (Fig. 4-IV), including *C*-pentosyl-apigenin *O*-*p*-coumaroylhexoside, *C*-pentosyl-apeignin *O*-feruloylhexoside, *C*-hexosyl-chrysoeriol *O*-feruloylhexoside *et al*., decreased levels were observed under 12 h UV-B irradiation and the majority of them slightly increased after more than 24 h treatment. However, di-glycosylated flavonoids, including *C*/*C* or *C*/*O* di-glycosylation, without any acylation (Fig. 4-VI), such as *C*-pentosyl-apigenin *O*-hexoside and *C*-pentosyl-chrysoeriol *O*-hexoside, exhibited decreases levels during UV-B irradiation.

### Analysis of differentially expressed genes involved in amino acid metabolic pathways in *G*. *uralensis* leaves under UV-B irradiation

**Figure 5:**
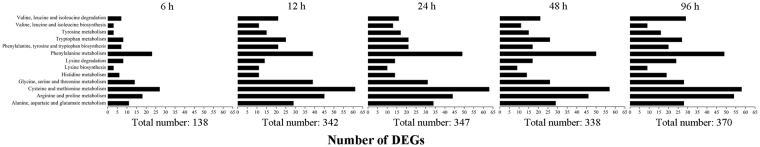
The numbers of DEGs related to amino acid metabolism in *Glycyrrhiza uralensis* leaves after UV-B exposure. A total of 138, 342, 347, 338, and 370 DEGs are related to amino acid metabolism in leaves under UV-B irradiation for 6, 12, 24, 48, and 96 h, respectively, based on the KEGG database.

Among the functionally annotated unigenes, differentially expressed genes (DEGs) related to amino acid metabolism were identified from the Kyoto Encyclopedia of Genes and Genomes (KEGG) annotation based on two criteria: *P* < 0.05 and |log_2_ (fold change)| > 1.0. The total number of DEGs involved in various amino acid metabolic pathways increased dramatically after 12 h or more of UV-B irradiation (Fig. 5, Supplementary Table S6), indicating that different periods of UV-B irradiation induce responses related to amino acid metabolism at the transcriptomic level in *G*. *uralensis*. As shown in Fig. 5, there were more DEGs involved in ‘Arg and Pro metabolism’ and ‘Cys and Met’ than in other categories. Also, there were more DEGs involved in Phe metabolism, which is closely related to secondary metabolism, than in most other metabolic pathways under UV-B treatment. These results suggest that UV-B irradiation activates the amino acid metabolism in *G*. *uralensis* leaves, as hundreds of DEGs responded to UV-B irradiation.

**Figure 6:**
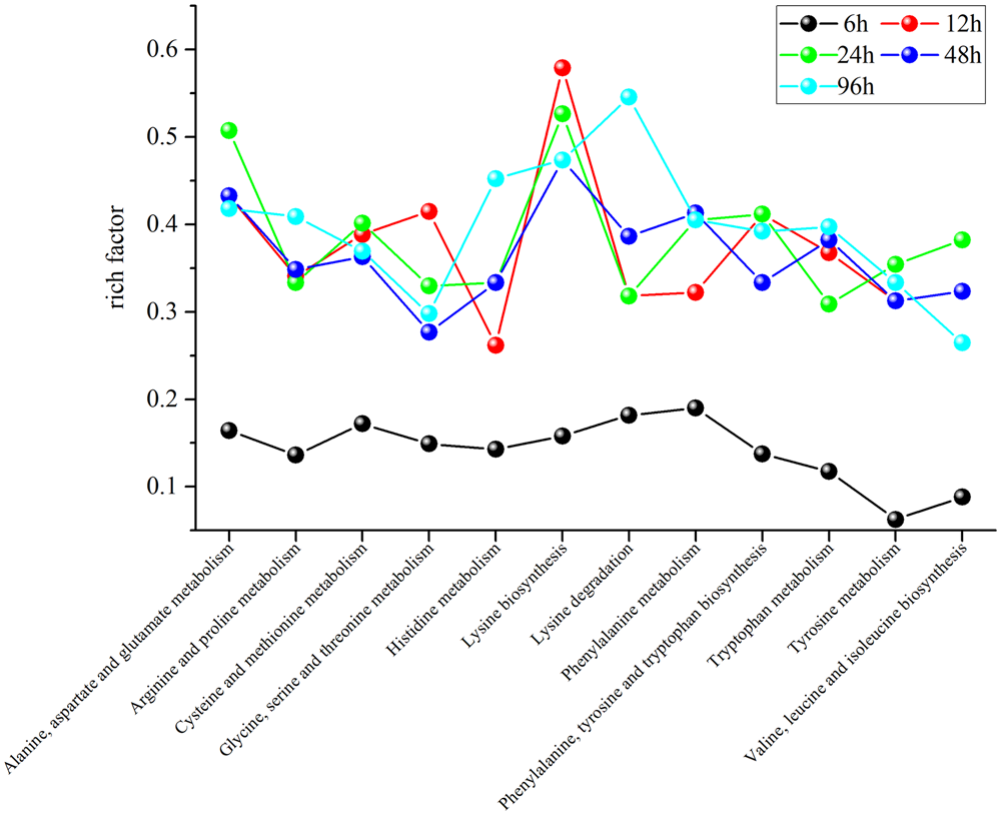
KEGG pathway enrichment analysis of various amino acid metabolic pathways in *Glycyrrhiza uralensis* leaves after UV-B exposure. The rich factors represent the ratio of the numbers of DEGs annotated in a certain pathway to the number of all genes mapped to this pathway. The higher the rich factor values, the more intensive the pathway.

**Figure 7:**
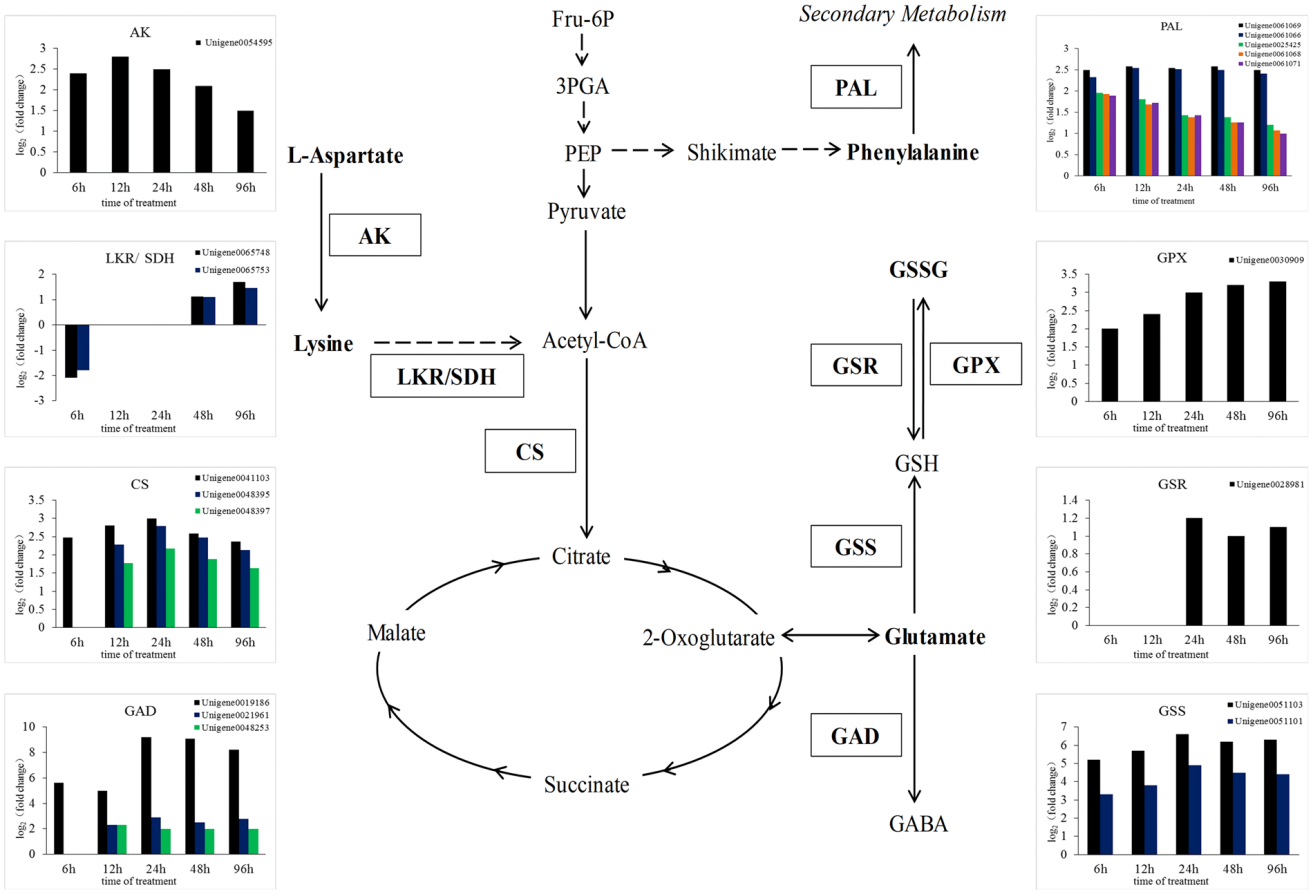
Modulation of aspartate, lysine, and glutamate metabolism combined with gene expression in *Glycyrrhiza uralensis* leaves under UV-B irradiation. Metabolites in bold were detected, and abbreviations in boxes indicate enzymes catalyzing the reactions. The expression patterns of unigenes encoding these enzymes are shown in histograms on both sides, and the lack of a column indicates not detected. The expression levels of unigenes are shown, and elevated expression levels were evaluated by log_2_ (fold change) > 1.0. Abbreviations for enzymes: AK, aspartate kinase; LKR/SDH, Lys-ketoglutarate reductase/saccharopine dehydrogenase; CS, citrate synthase; GAD, glutamate decarboxylase; GPX, glutathione peroxidase; GSR, glutathione-disulfide reductase; GSS, glutathione synthase; PAL, phenylalanine ammonia lyase. Abbreviations for metabolites: Fru-6P, fructose 6-phosphate; 3PGA, 3-phosphoglyceric acid; PEP, phosphoenolpyruvate; GABA, γ-aminobutyric acid; GSH, glutathione; GSSG, glutathione (oxidized).

In addition, the expression levels of members of the Lys biosynthesis pathway were highest among amino acid metabolic pathway genes at 12, 24, and 48 h of UV-B treatment (Fig. 6), suggesting that DEGs involved in Lys biosynthesis were highly enriched compared to other pathways, despite the relatively small number of genes. Also, expression of a unigene encoding aspartate kinase (AK), whose activity leads to Lys biosynthesis, increased during UV-B irradiation (Fig. 7). In addition, two unigenes encoding the enzyme Lys-ketoglutarate reductase (LKR)/saccharopine dehydrogenase (SDH), which is involved in Lys catabolism, were upregulated after 48 and 96 h of UV-B irradiation (Fig. 7). Among genes in the Lys degradation pathway, unigenes encoding citrate synthase (CS), catalyzing the formation of citrate, a TCA cycle intermediate, were upregulated under UV-B treatment (Fig. 7). Another amino acid closely related to the TCA cycle is Glu, whose metabolism plays an important role in plants. Among genes in the Glu metabolism pathway, we identified three *GAD* (glutamate decarboxylase) genes, encoding an enzyme that specifically catalyzes Glu to γ-aminobutyric acid (GABA), and two *GSS* (glutathione synthase) genes, which are involved in the production of GSH from Glu. *GAD* and *GSS* genes were both upregulated throughout treatment (Fig. 7), implying that Glu is most likely converted into GABA and GSH under UV-B irradiation in *G*. *uralensis* leaves. We also identified DEGs encoding glutathione peroxidase (GPX) and glutathione-disulfide reductase (GSR), which catalyze the conversion reactions between GSH and GSSG. The expression of the *GPX* genes was elevated at all five time points and that of *GSR* genes was elevated after 24 h or more of UV-B treatment (Fig. 7), suggesting that GSH plays a positive role in *G*. *uralensis* leaves after exposure to UV-B radiation.

### Analysis of DEGs involved in phenylpropanoid and flavonoid biosynthesis pathways in *G*. *uralensis* leaves in response to UV-B radiation

**Figure 8:**
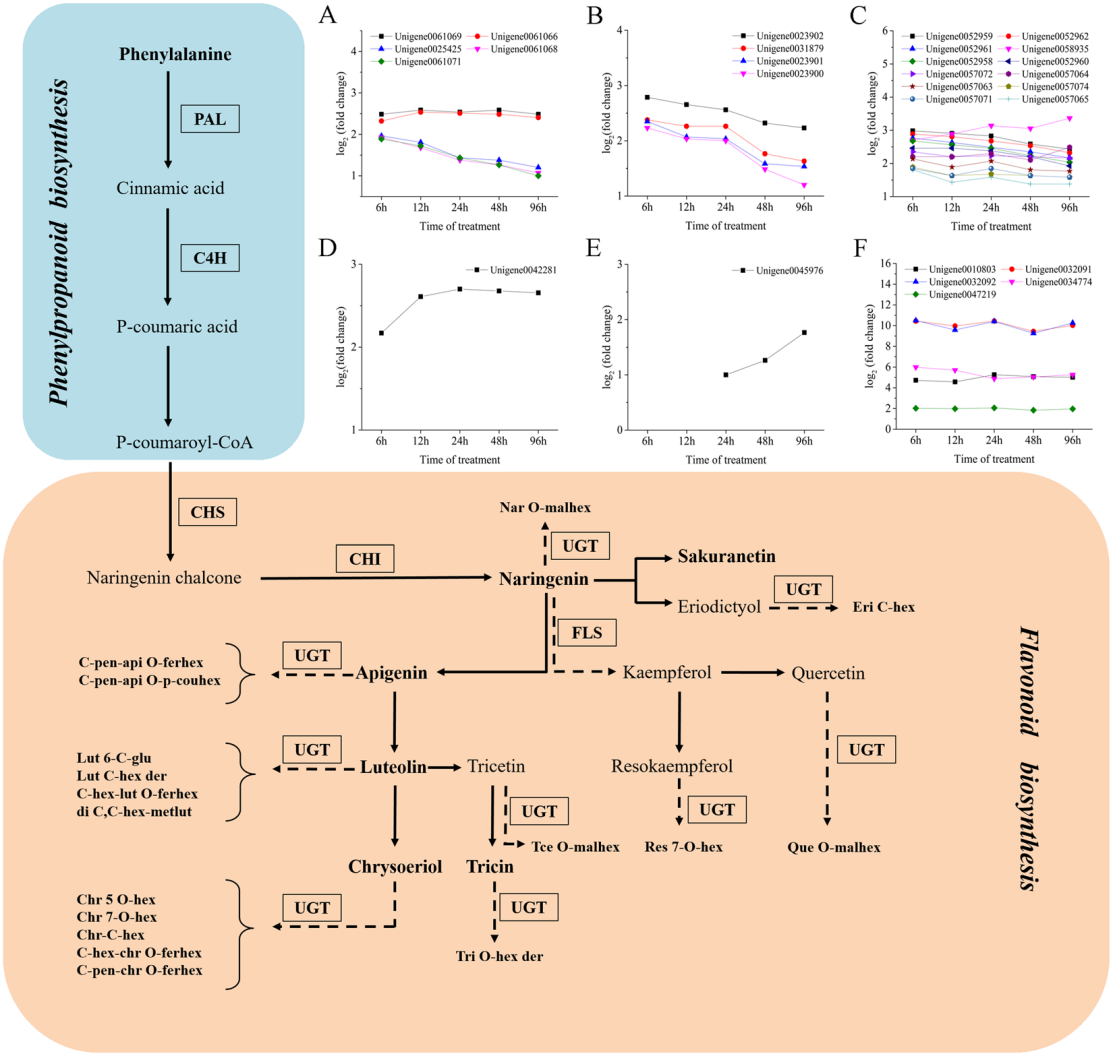
Modulation of phenylpropanoid and flavonoid biosynthesis pathways combined with gene expression in *Glycyrrhiza uralensis* leaves under UV-B irradiation. Metabolites in bold were detected, and abbreviations in boxes indicate enzymes catalyzing the reactions. The expression patterns of unigenes encoding these enzymes are shown in line charts in the upper right (A, *PAL*; B, *C4H*; C, *CHS*; D, *CHI*; E, *FLS*; F, *UGT*), and the lack of points indicates not detected. The expression levels of unigenes are shown, and elevated expression levels were evaluated by log_2_ (fold change) > 1.0. Abbreviations for enzymes: PAL, phenylalanine ammonia-lyase; C4H, cinnamate 4-hydroxylase; CHS, chalcone synthase; CHI, chalcone isomerase; FLS, flavonol synthase; UGT, UDP-glycosyltransferase. Abbreviations for metabolites: Nar *O*-malhex, Naringenin *O*-malonylhexoside; Eri *C*-hex, Eriodictyol *C*-hexoside; *C*-pen-api *O*-ferhex, *C*-pentosyl-apigenin *O*-feruloylhexoside; *C*-pen-api *O*-p-couhex, *C*-pentosyl-apigenin *O*-*p*-coumaroylhexoside; Lut 6-*C*-glu, Luteolin 6-*C*-glucoside; Lut *C*-hex der, Luteolin *C*-hexoside derivative; *C*-hex-lut *O*-ferhex, *C*-hexosyl-luteolin *O*-feruloylhexoside; di *C*, *C*-hex-metlut, di-*C*,*C*-hexosyl-methylluteolin; Chr 5 *O*-hex, Chrysoeriol 5-*O*-hexoside; Chr 7-*O*-hex, Chrysoeriol 7-*O*-hexoside; Chr-*C*-hex, Chrysoeriol *C*-hexoside; *C*-hex-chr *O*-ferhex, *C*-hexosyl-chrysoeriol *O*-feruloylhexoside; *C*-pen-chr *O*-ferhex, *C*-pentosyl-chrysoeriol *O*-feruloylhexoside; Tce *O*-malhex, Tricetin *O*-malonylhexoside; Tri *O*-hex der, Tricin *O*-hexoside derivative; Res 7-*O*-hex, Resokaempferol 7-*O*-hexoside; Que *O*-malhex, Quercetin *O*-malonylhexoside.

In the present work, we identified 18 candidate unigenes involved in phenylpropanoid and flavonoid biosynthesis, as shown in Table 2. Five DEGs encoding phenylalanine ammonia lyase (PAL), the rate-limiting enzyme in the phenylpropanoid biosynthesis pathway, were upregulated by UV-B treatment, as were four DEGs encoding cinnamate 4-hydroxylase (C4H), which catalyzes the conversion of cinnamic acids into *p*-coumaric acid (Fig. 8). Among genes in the flavonoid biosynthesis pathways, seven *CHS* (chalcone synthase) genes and one *CHI* (chalcone isomerase) gene were more highly expressed in *G*. *uralensis* leaves after UV-B irradiation compared to the controls; these enzymes are involved in reactions that form flavanones such as naringenin (Fig. 8). Finally, the expression of one unigene encoding flavonol synthase (FLS) increased in *G*. *uralensis* plants after UV-B radiation for 24 h or more (Fig. 8).

**Table 2:** DGEs involved in phenylpropanoid and flavonoids biosynthesis in response to UV-B radiation.

Gene ID	Gene annotation	Fold change				
		6 h	12 h	24 h	48 h	96 h
Unigene0061069	phenylalanine ammonialyase (PAL)	5.62	5.98	5.82	5.98	5.62
Unigene0061066	phenylalanine ammonialyase (PAL)	4.99	5.82	5.70	5.62	5.31
Unigene0025425	phenylalanine ammonialyase (PAL)	3.89	3.51	2.69	2.60	2.30
Unigene0061068	phenylalanine ammonialyase (PAL)	3.81	3.20	2.60	2.39	2.10
Unigene0061071	phenylalanine ammonialyase (PAL)	3.71	3.29	2.69	2.39	2.00
Unigene0023902	cinnamate-4-hydroxylase(C4H)	6.92	6.32	5.90	4.99	4.69
Unigene0031879	cinnamate-4-hydroxylase(C4H)	5.21	4.79	4.79	3.41	3.10
Unigene0023901	cinnamate-4-hydroxylase(C4H)	5.10	4.20	4.11	2.99	2.91
Unigene0023900	cinnamate-4-hydroxylase(C4H)	4.69	4.11	4.00	2.81	2.30
Unigene0052959	chalcone synthase (CHS)	7.89	7.52	7.11	5.98	5.39
Unigene0052962	chalcone synthase (CHS)	7.41	7.01	6.41	5.82	4.99
Unigene0052958	chalcone synthase (CHS)	6.41	5.90	5.50	4.69	4.11
Unigene0057072	chalcone synthase (CHS)	5.10	4.59	4.69	4.59	4.50
Unigene0057064	chalcone synthase (CHS)	4.59	4.59	4.89	4.29	5.62
Unigene0057063	chalcone synthase (CHS)	4.41	3.71	4.20	3.51	3.41
Unigene0057074	chalcone synthase (CHS)	3.71	3.10	3.20	3.10	2.99
Unigene0042281	chalcone isomerase (CHI)	4.50	6.11	6.50	6.41	6.32
Unigene0045976	flavonol synthase (FLS)	—	—	2.00	2.39	3.41

We also detected five unigenes annotated as *UGTs* (UDP-glycosyltransferase), which might encode enzymes catalyzing the glycosylation reaction of various flavonoids (Table 3). The five *UGTs* identified by transcriptome analysis were upregulated under UV-B treatment (Fig. 8). Taken together, these results indicate that all DEGs related to the phenylpropanoid and flavonoid biosynthesis pathways identified in this study were upregulated after exposure to UV-B.

**Table 3:** DGEs related to flavonoids glycosylation under UV-B radiation.

Gene ID	Gene annotation	fold change				
		6 h	12 h	24 h	48 h	96 h
Unigene0010803	UDP-glycosyltransferase 85A3-like	26.54	23.75	38.32	33.82	32.45
Unigene0032091	UDP-glycosyltransferase 74B1-like	1389.16	1002.93	1389.16	694.58	1045.52
Unigene0032092	UDP-glycosyltransferase 74B1	1448.15	770.69	1360.57	613.11	1234.75
Unigene0034774	UDP-glycosyltransferase 72E2	63.12	51.98	29.45	33.13	38.32
Unigene0047219	UDP-glycosyltransferase 89B1	4.06	3.94	4.17	3.58	3.92

### Quantitative Real-Time PCR (qRT-PCR) Validation of DEGs from RNA-Seq

**Figure 9:**
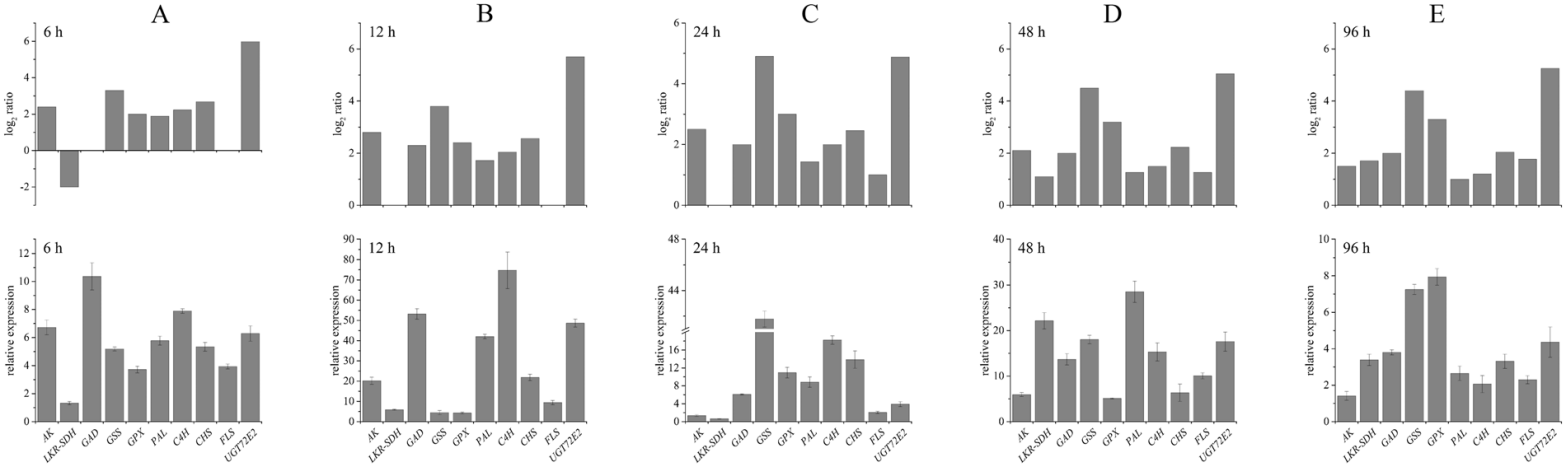
Validation of expression patterns of ten DEGs by qRT-PCR assay. Expression patterns of 10 DEGs involved in amino acid metabolism (6 DEGs) and flavonoids biosynthesis pathways (4 DEGs) by RNA-Seq (histograms located above) and qRT-PCR (histogram located blow) in *Glycyrrhiza uralensis* leaves after UV-B irradiation for 6 h (**A**), 12 h (**B**), 24 h (**C**), 48 h (**D**), and 96 h (**E**).

The differential expression levels of DEGs were confirmed by qRT-PCR, and 10 unigenes involved in amino acid metabolism (6 DEGs) and flavonoid biosynthesis pathways (4 DEGs) were selected to validate the RNA-Seq results, including *AK* (Unigene0054595), *LKR/SDH* (Unigene0065748), *GAD* (Unigene0048253), *GSS* (Unigene0051101), *GPX* (Unigene0030909), *PAL* (Unigene0061071), *C4H* (Unigene0023900), *FLS* (Unigene0045976), and *UGT72E2* (Unigene0034774). The qRT-PCR results showed that seven of these genes showed up-regulation after 6 h UV-B irradiation, consistent with the RNA-Seq data (Fig. 9A). Eight and nine of the ten genes were up-regulated after 12 h and 24 h UV-B irradiation, respectively, which was consistent with the increased expression of these unigenes in the RNA-Seq data. Inconsistent results for a few unigenes, including *LKR/SDH* at 12 and 24 h, and *FLS* at 12 h of treatment, were likely due to the fact that they were not detected in the RNA-Seq data (Fig. 9B,C). All ten genes were upregulated after 48 h and 96 h UV-B irradiation, in line with the RNA-Seq data (Fig. 9D,E). Thus, the RNA-Seq results were reliable for the identification and measurement of expression of DEGs involved in amino acid metabolism and flavonoid biosynthesis pathways in *G*. *uralensis* leaves under different durations of UV-B radiation.

## Discussion

*G*. *uralensis* is one of the oldest Chinese medicinal herbs, and has high levels of bioactive phytochemicals^22,23^ and multiple pharmacological properties^24–27^. However, previous studies have mainly focused on the roots of this species^40–43^. In order to investigate the variations of metabolites in *G*. *uralensis* leaves under UV-B irradiation, we performed non-targeted metabolic analysis. We found that UV-B irradiation has a marked effect on the metabolic profiles of *G*. *uralensis* leaves and metabolite levels in the leaves changed with different durations of irradiation. In order to explore the mechanism underlying the varied metabolites in *G*. *uralensis* leaves in response to UV-B treatment, we examined the changes in primary and secondary metabolites and analyzed DEGs involved in corresponding metabolic pathways under UV-B irradiation. We found that unigenes related to amino acid metabolism and flavonoid biosynthesis pathways were activated in response to UV-B radiation and that these variations at the transcript level ultimately led to changes in metabolite profiles.

Amino acids are major primary metabolites in plants and, in addition to forming proteins, amino acids can be catabolized into intermediates of the TCA cycle for energy generation^44,45^. In addition, amino acids can function as the precursors of secondary metabolites that can protect plants from various stresses^46^. Amino acid metabolic pathways play important roles in regulating plant growth and development^44,47^ as well as stress-related metabolism^48,49^. The conversion of amino acids to TCA cycle intermediates for energy generation^45,47^ involved in stress response implies resource reallocation of plants in the face of stress^48^. In addition, stress-associated defensive metabolites synthesized from amino acid metabolic pathways demonstrate the vital roles of amino acid metabolism in response to stresses in plants^48,50^. Previous studies found that several amino acids, including Pro, Ser, Leu, Ile, and Glu were accumulated in plants following exposure to high-level UV-B irradiation^51^, and amino acid metabolism-related pathways were enhanced, such as shikimate metabolic pathway^10^ and leucine/isoleucine related pathways^52^ in leaves of *C*. *terniflora* exposed to UV-B irradiation. However, the specific mechanism underlying the amino acids metabolism that results in different accumulations of amino acids in *G*. *uralensis* leaves under UV-B irradiation has not yet been characterized. In this study, variations of amino acids that responded to different durations of UV-B irradiation were found in *G*. *uralensis* leaves, including Lys, Asp, Glu and Phe, which were closely linked with other metabolic pathways for regulating amino acid metabolism and deploying defense responses.

**Figure 10:**
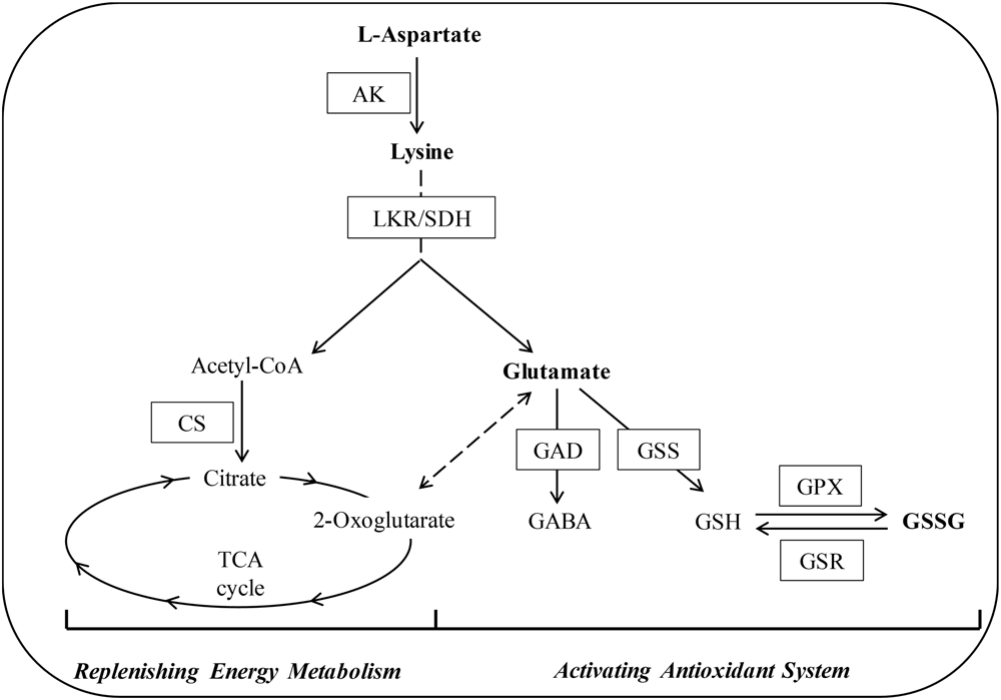
Schematic diagram of proposed regulatory metabolic network among Asp, Lys and Glu in *G*. *uralensis leaves* in response to UV-B irradiation. Metabolites in bold were detected, and abbreviations in boxes indicate enzymes catalyzing the reactions. Solid lines represent one-step reactions, and dashed lines represent multi-step reactions.

Particularly, the contents of Lys increased significantly at four time points of UV-B irradiation (*P* < 0.05, Supplementary Table S5) compared to the controls, which implies that *G*. *uralensis* leaves tend to accumulate Lys when exposed to UV-B radiation. Lys has been suggested to be involved in plant stress responses^44^, which indicates that the accumulated Lys in *G*. *uralensis* leaves after UV-B treatment may be associated with UV responses. As the precursor of Lys, Asp levels decreased slightly in *G*. *uralensis* leaves after UV-B irradiation for different lengths of time. Moreover, the expression of unigenes encoding AK increased after exposure to UV-B irradiation (Fig. 7), suggesting that the metabolic flux was likely directed into Lys biosynthesis through the Asp-family pathway, which has been demonstrated to link with energy and stress regulation^44^. Then, the accumulated Lys in *G*. *uralensis* leaves after UV-B exposure might be degraded to replenish TCA cycle metabolites due to the expression of *LKR*/*SDH*, which encodes a bifunctional polypeptide converting Lys into the energy-associated TCA cycle metabolite acetyl-CoA^53^ in response to energy starvation upon stress conditions^44^, was up-regulated after UV-B irradiation for more than 48 h. The enhanced expression of *LKR/SDH*, which is involved in Lys catabolism, in response to stress has been reported^46,54^. Accordingly, the UV-induced LKR/SDH in *G*. *uralensis* leaves likely contributes to Lys catabolism, which may play an important role in stress response under UV-B irradiation. Acetyl-CoA reacts with oxaloacetate to form citrate through CS, which was upregulated by UV-B irradiation (Fig. 7), in the first step of the TCA cycle. This further suggests that Lys catabolism into acetyl-CoA can likely contribute to the TCA cycle to supplement the energy lost in *G*. *uralensis* leaves in response to UV-B stress. It is suggested that stress-induced Lys catabolism generates Glu, which is the precursor of stress-related metabolites and therefore can contribute to plant responses to environmental stress^50,53^. Glu functions as a plant signaling molecule playing vital roles in regulating plant growth and defense responses^10,50–57^. In the current study, Glu levels increased after 12 and 24 h of UV-B irradiation compared to the controls, followed by a slight decrease at 48 and 96 h of treatment. The declining levels of Glu might play an important role in maintaining the redox state through conversion into GSH and GABA in *G*. *uralensis* leaves under UV-B irradiation. One the one hand, Glu combines with cysteine and glycine to produce GSH via enzymatic reactions catalyzed by the GSS enzyme. GSH plays a role in maintaining the redox balance in plants and helping them cope with oxidative stress^58–61^. The increased expression of unigenes encoding GSSs after UV-B exposure suggests that Glu generates GSH under UV-B irradiation in *G*. *uralensis* leaves. The conversion reaction between the reduced form of glutathione (GSH) and the oxidized form of glutathione (GSSG), which function as a redox couple in plants to help plants maintain a relatively stable GSH/GSSG ratio^62^, via GPX and GSR. The antioxidant marker gene *GPX* is upregulated under abiotic stress and is thought to be involved in ROS scavenging in *Panax ginseng*^63^. GSR activity was induced by oxidative stress caused by UV-B radiation in transgenic tobacco plants, thereby giving the plants increased resistance to UV-B stress^64–66^. The increased expression of unigenes encoding GPX and GSR indicates that ROS or other free radicals might be generated in *G*. *uralensis* leaves exposed to UV-B radiation. We detected increased levels of the oxidized form of glutathione (GSSG) via metabolic profiling (Supplementary Table S5), corresponding to the increased expression of unigenes encoding the related enzymes. On the other hand, GABA responds to various abiotic stresses in plants including drought, salt, heat, cold, low oxygen levels, and ROS^67–70^. The enzyme GAD catalyzes Glu into GABA which is the first step of the GABA shunt and the activity of GAD affects the metabolism of the GABA shunt^71,72^. In our results, unigenes encoding GAD were upregulated in *G*. *uralensis* leaves under UV-B irradiation (Fig. 7), suggesting that the gradual decrease in Glu levels was likely due to its catalysis into GABA to mitigate the oxidative stress caused by UV-B irradiation. Accordingly, the UV-induced Glu derived from Lys catabolism plays a vital role in *G*. *uralensis* leaves in response to UV-B radiation through its catabolism into GABA and GSH in order to reduce oxidative stress caused by UV-B irradiation. In general, Lys, Asp, and Glu are closely related to each other to form a regulatory metabolic network in *G*. *uralensis* leaves in response to UV-B irradiation through replenishing energy-associated TCA cycle metabolites and forming antioxidant compounds (Fig. 10).

In addition to primary metabolism, several amino acids contribute to biosynthesis of secondary metabolites, such as flavonoids, upon UV-B irradiation^46^. Especially, Phe serves as the entry compound of phenylpropanoid metabolism, which leads to the biosynthesis of flavonoids in plants^73^. In our results, increased levels of Phe in *G*. *uralensis* leaves were observed after UV-B irradiation for 12 or 24 h, and slight decreases were observed after UV treatment for more than 48 h. Moreover, unigenes encoding PAL and C4H, two key enzymes involved in the phenylpropanoid biosynthesis pathway, were upregulated after exposure to UV-B in *G*. *uralensis* leaves (Fig. 8). The elevated expression levels of PAL and C4H might indicate that UV-B irradiation promotes the conversion of Phe into flavonoids by stimulating PAL and C4H in *G*. *uralensis* leaves under UV-B irradiation. These results imply that phenylalanine metabolism is pushed towards the biosynthesis of flavonoids, which will help protect *G*. *uralensis* from UV-B irradiation.

Extensive works have been carried out to study the accumulation of flavonoids in plants under UV and its function as absorbing compounds^74^ or free radical scavengers to reduce the damage of plants^75–79^. Recently, UV-protective function of flavone *O*-glucosides were reported^80^, and glycosylated flavonols significantly increased after UV exposure in plants^9^. However, as of now, there is no report on either the effect of UV irradiation on leaves, or its flavonoids, especially glycosylated flavonoids accumulations in *G*. *uralensis* leaves under UV stress. In the current study, we detected kinds of flavonoids in *G*. *uralensis* leaves and found that their variation patterns during the time course of UV-B irradiation were related to their respective modification groups. Many of them were glycosylated flavonoids such as mono-*C*/*O* glycosylated flavonoids and di-*C*, *C*-glycosyl or di-*C*, *O* glycosylated flavonoids. Flavonoids commonly occur in plants as glycosides, since glycosylation can increase the water solubility of flavonoids and protect their reactive groups from oxidation by free radicals^16,73^. In addition, glycosylated flavonoids can be further acylated by aliphatic or aromatic acetyl groups^6,81^, such as the malonylated flavonoids and those with feruloyl or coumaroyl modifications detected in our study. In particular, we found that flavonoid glycosides with modifications of distinct groups in *G*. *uralensis* leaves under UV-B irradiation displayed distinct variation patterns during the treatment periods (Fig. 4), suggesting that flavonoids with different substituent groups might play different roles in the response to UV-B irradiation in *G*. *uralensis* leaves. The diverse variation patterns (according to the different modification groups) might also indicate that the activities of flavonoids are affected by different types and positions of glycosylation^73^. The glycosylated flavonoids have been reported in species such as maize, wheat, and rice^39,81–83^, and *C*-glycosides of flavones such as apigenin and/or luteolin were suggested to act as antioxidants, insect feeding attractants, and UV-protective pigments^82,84^. However, the functions of most glycosylated flavonoids detected in this work and the correlation between their functional mechanisms and substitutions of groups in *G*. *uralensis* leaves in the response to UV-B irradiation remain to be explored. Furthermore, flavonoids with different chemical structures differ in their antioxidant activity, which has been mainly ascribed to the hydroxyl groups substituent to them, and the ortho-dihydroxylated B-ring flavonoids have been suggested as important potential radical-scavenging compounds^85,86^. In this work, we observed an increase in the levels of flavonoids with two hydroxyl groups attached to B-rings, such as quercetin *O*-malonylhexoside. Numerous studies have demonstrated that ortho-dihydroxylated B-ring flavonoids such as quercetin and its derivatives, accumulate in plants under stresses, including UV irradiation, and function as inhibitors of ROS generation and as ROS scavengers^15–17,87–91^. The increased dihydroxy B-ring-substituted flavonoids detected in our study might scavenge the ROS in *G*. *uralensis* leaves to protect the plant from oxidative stress caused by UV-B irradiation. In particular, we found that the levels of apigenin and several of its glycosides exhibited an upward trend in this study, nevertheless the levels of apigenin glycoside and its derivatives changed little under increased irradiation in other species^16,92^. Apigenin is a common flavonoid, and along with several of its *C*-glycosylated derivatives, has anti-cancer and anti-inflammatory activities and inhibit RNA viruses^93–97^. In the present study, the level of apigenin increased sharply in *G*. *uralensis* leaves treated with UV-B, suggesting that *G*. *uralensis* preferentially synthesizes apigenin in leaves under UV-B irradiation. Therefore, *G*. *uralensis* leaves irradiated by UV-B may have increased pharmaceutical activity for prevention and treatment of human disease. In addition, levels of other flavonoids also increased significantly after UV-B irradiation, such as naringenin, tricetin *O*-malonylhexoside, di-*C*,*C*-hexosyl-methylluteolin, etc. The levels of these flavonoids were elevated in UV-B-treated *G*. *uralensis* leaves, but the exact roles of these compounds upon UV-B stress response remain to be investigated.

Unigenes encoding the key enzymes in flavonoid biosynthesis and glycosylation were upregulated in *G*. *uralensis* leaves in response to UV-B treatment, including *CHS*, *FLS*, and *UGT*. UGTs mediate a major modification of secondary metabolites, i.e. glycosylation^98^. Many flavonoids detected in our study were glycosylated by UGTs, although the exact functions of the respective UGTs have not been determined. The qRT-PCR results were consistent with the expression pattern of unigenes detected in RNA-Seq data. The upregulation of these genes implies that UV-B irradiation induced flavonoid biosynthesis pathways and the related modifications of glycosylation in *G*. *uralensis* leaves.

We performed comprehensive metabolic profiling to investigate the variations of primary and secondary metabolites in *G*. *uralensis* leaves under various durations of UV-B irradiation. We identified/annotated 54 metabolites in *G*. *uralensis* leaves during the course of UV-B irradiation by using an LC-MS/MS system. The altered levels of various metabolites, primarily including those involved in amino acid metabolism and flavonoid biosynthesis pathways, together with the variation in the transcript levels of related unigenes, shed light on the potential interaction of amino acid metabolism with plant energy and secondary metabolism in *G*. *uralensis* leaves in response to UV-B irradiation. Our results increase our understanding of the metabolic regulation of *G*. *uralensis* leaves in response to UV-B irradiation and pave the way for further dissection of metabolic pathways in the pharmaceutical plant *G*. *uralensis* under adverse conditions.

## Methods

### Plant materials

*Glycyrrhiza uralensis* (Fisch.) seeds were collected from the Liquorice Planting Base in Yanchi County, Ningxia province. Mature, plump seeds were surface sterilized in concentrated H_2_SO_4_ for 25 minutes, washed for 2 h with tap water, and sown in pots (10 cm * 10 cm * 10 cm) filled with moistened sandy soil. The pots were placed in a growth chamber with 20–30% relative humidity, 145 μmol m^−2^ s^−1^ photosynthetically active radiation (PAR), and 27/24 °C during the light/dark (12 h/12 h) periods, respectively.

### Experimental design and UV-B radiation treatment

At 60 days after germination, healthy seedlings were selected and randomly divided into four groups for metabolome analysis and six groups for transcriptome analysis. For metabolome analysis, each group had three replicates, each with two plants. The control groups of plants were exposed to continuous light supplied by T8 36 W, 120 cm fluorescent tubes (Foshan Electrical Co. Ltd. Foshan, China). The PAR was 145 μmol m^−2^ s^−1^ measured by a digital illuminometer ST-80C (Photoelectric Instrument Factory of Beijing Normal University, Beijing, China). The treatment groups of plants were continually exposed to supplemental UV-B for 12, 24, 48 and 96 h. For transcriptome analysis, each group had three replicates, each with two plants. The control groups of plants were also exposed to continuous light without any UV irradiation. The treatment groups of plants were continually irradiated for 6, 12, 24, 48, and 96 h. All UV-B-irradiated plants were transferred to another growth chamber with the same growth conditions as the control. UV-B was provided by 308–310 nm UV-B fluorescent tubes (Beijing Photoelectric Instrument Co. Ltd., Beijing, China). The intensity of UV-B radiation was set at 0.024 W/m^2^, which matches the proportion of UV-B radiation in sunlight in the field from May to July in Ningxia province, China. A UV-B radiometer (Photoelectric Instrument Factory of Beijing Normal University, Beijing, China) was used to measure the intensity of UV radiation. To filter out shorter wavelengths of light (λ ≤ 280 nm, such as UV-C), 1.5-mm thick cellulose diacetate film (E. I. du Pont de Nemours and Company, USA) was placed under the fluorescent tubes before the irradiation was started.

### Chemicals

The solvents used for extraction, including acetonitrile, acetic acid, and methanol, were of HPLC-grade (Merck, Darmstadt, Germany). The water used in this study was doubly deionized using a MilliQ ULTRA purification system (Millipore, Vimodrone, Italy). Lidocaine, which was used as an internal standard, was purchased from Shanghai New Asiatic Pharmaceuticals Co., Ltd. (http://www.xinyapharm.com/). All authentic standards were purchased from Sigma-Aldrich, USA (www.sigmaaldrich.com/united-states.html). Standard stock solutions of flavonoids and amino acids were prepared using methanol and deionized water as the respective solvents and stored at −20 °C.

### Sample preparation and extraction

All leaf samples were collected from 10:00 to 11:00 am, immediately frozen in liquid nitrogen, ground into fine powder in liquid nitrogen, and vacuum freeze-dried for 24 h. Approximately 100 mg of each sample was combined with 1.0 ml of solvent comprising 70% aqueous methanol with 0.1 mg/l lidocaine and extracted overnight at 4 °C. After centrifugation at 10,000 g for 10 min, the supernatants were filtered (SCAA-104, 0.22 μm pore size; Anpel, Shanghai, China, http://www.anpel.com.cn/) before LC-MS analysis^35^.

### Metabolite analysis by LC-MS/MS

Leaf samples were analyzed via HPLC-ESI-QTOF-MS/MS system (6520B, Agilent, USA), and fragmentation patterns were obtained in the targeted MS^2^ mode. The data were processed using MassHunter Qualitative Analysis software^35^ (Agilent Technologies, Barcelona, Spain). Multiple reaction monitoring (MRM) was performed using the LC-ESI-Q TRAP-MS/MS (4000Q TRAP, ABI, USA) for quantification of metabolites. Data acquisition, curves calibration, peak integration, and calculation were implemented with Analyst 1.6 software (AB Sciex). Data processing and the analytical conditions were as described previously^35^. Qualitative and quantitative chromatographic parameters were the same: The HPLC column was a shim-pack VP-ODS C18 (pore size 5.0 μm, length 2 × 150 mm); the column temperature was set at 40 °C; the solvent system was selected as water (0.04% acetic acid added): acetonitrile (0.04% acetic acid added). The gradient program was as follows: 100:0 V/V at 0 min, 5:95 V/V at 20.0 min, 5:95 V/V at 22.0 min, 95:5 V/V at 22.1 min, 95:5 V/V at 28.0 min; the flow rate was 0.25 ml min^−1^; and the volume of injection was 5 μl. Flavonoid quantification was performed by calculating each individual peak area and comparing the data with standard curves acquired from authentic flavonoid standards, including apigenin 6-*C*-glucoside, apigenin 7-*O*-glucoside, luteolin 7-*O*-glucoside, chrysoeriol, naringenin 7-*O*-glucoside, naringenin 8-*C*-glucoside, and quercetin 3-*O*-glucoside. To quantify the amino acids, the area of each individual peak was calculated and compared to the standard curves obtained from authentic standard tryptophan (Supplementary Table S4). All standard curves were constructed using authentic standards at four different concentrations under the same analytical conditions.

### Statistical analyses

Principal component analysis (PCA) was performed with R (www.r-project.org/) using the Pareto scaling method to obtain grouping information for non-targeted metabolomics data. Seventeen amino acids and 36 flavonoids were subjected to hierarchical clustering analysis via HemI (http://hemi.biocuckoo.org/) to visualize changes in metabolite profiles.

### Sample collection, RNA extraction, and RNA-sequencing for transcriptome analysis

*G*. *uralensis* leaves treated with UV-B irradiation for 6, 12, 24, 48, and 96 h, as well as the control, were collected for RNA extraction. Each treatment was repeated with three biological replicates. Total RNA was extracted from seedlings using an RNAprep Pure Plant kit (Tiangen, China) according to the manufacturer’s instructions. After enrichment with oligo(dT) beads, the fragmented mRNA was reverse transcribed into cDNA with random primers. Second-strand cDNA was synthesized using DNA polymerase I, RNase H, dNTP, and buffer. The cDNA fragments were purified with a QiaQuick PCR Extraction kit, end repaired, poly(A) tailed, and ligated to Illumina sequencing adapters. The ligation products were size selected by agarose gel electrophoresis, PCR amplified, and sequenced using an Illumina HiSeq. 4000 system from Gene Denovo Biotechnology Co. (Guangzhou, China). The metrics of RNA sequencing analysis are provided in Supplementary Table S7.

### Analysis of differentially expressed genes

Unigene expression levels were calculated and normalized to RPKM (Reads Per kb per Million reads)^99^. To annotate the unigenes, the BLASTx program (http://www.ncbi.nlm.nih.gov/BLAST/) was used with an *E*-value threshold of 1e-5 against the NCBI non-redundant protein (Nr) database (http://www.ncbi.nlm.nih.gov), the Swiss-Prot protein database (http://www.expasy.ch/sprot), the KEGG database (http://www.genome.jp/kegg), and the COG/KOG database (http://www.ncbi.nlm.nih.gov/COG). Protein functional annotations were then obtained according to the best alignment results. To identify differentially expressed genes (DEGs) across groups, the edgeR package (http://www.r-project.org/) was used. Genes with a fold change ≥ 2 and a false discovery rate (FDR) < 0.05 were defined as significant DEGs.

### Quantitative real-time polymerase chain reaction (qRT-PCR)

The total RNA was extracted from samples collected at the same time points (0, 6, 12, 24, 48, and 96 h; 0-h sample was used as the control) as the ones collected for RNA-Seq analysis, reverse-transcribed (Takara, http://ww.takara-bio.com/), and subjected to PCR in a BioRad CFX96 Real-Time System according to the manufacturer’s instructions. The specific primers for the ten selected genes were designed after BLASTing the sequences from of our RNA-Seq with the CDS of *Glycyrrhiza uralensis* genome^100^ (http://ngs-data-archive.psc.riken.jp/Gur-genome/blast.pl). Sequences of primers and the identities of the BLAST hits are provided in Supplementary Table S8. The *G*. *uralensis* actin gene (*Guactin*, GeneBank: EU 190972) was used as an internal reference standard, and the relative expression levels were calculated using the 2^*−ΔΔCT*^ method^101^.

### Data availability

All data generated or analysed during this study are included in this published article and its Supplementary Information files.

## Electronic supplementary material

Supplementary Information

Dataset 1

Dataset 2

Dataset 3

Dataset 4

Dataset 5

Dataset 6
