# Supplementary material for: Measurement of metabolite variations and analysis of related gene expression in Chinese liquorice (Glycyrrhiza uralensis) plants under UV-B irradiation
Source: Sci Rep. 2018 Apr 18;8:6144. doi: 10.1038/s41598-018-24284-4 (PMC5906665; doi:10.1038/s41598-018-24284-4)
Supplement: Supplementary file 1 — Supplementary Information [file 41598_2018_24284_MOESM1_ESM.pdf]

Supplementary Information

Measurement of metabolite variations and analysis of related gene expression in Chinese liquorice (*Glycyrrhiza uralensis*) plants under UV-B irradiation

Xiao Zhang[+], Xiaoli Ding[+], Yaxi Ji, Shouchuang Wang, Yingying Chen, Jie Luo, Yingbai Shen[*], Li Peng[*]

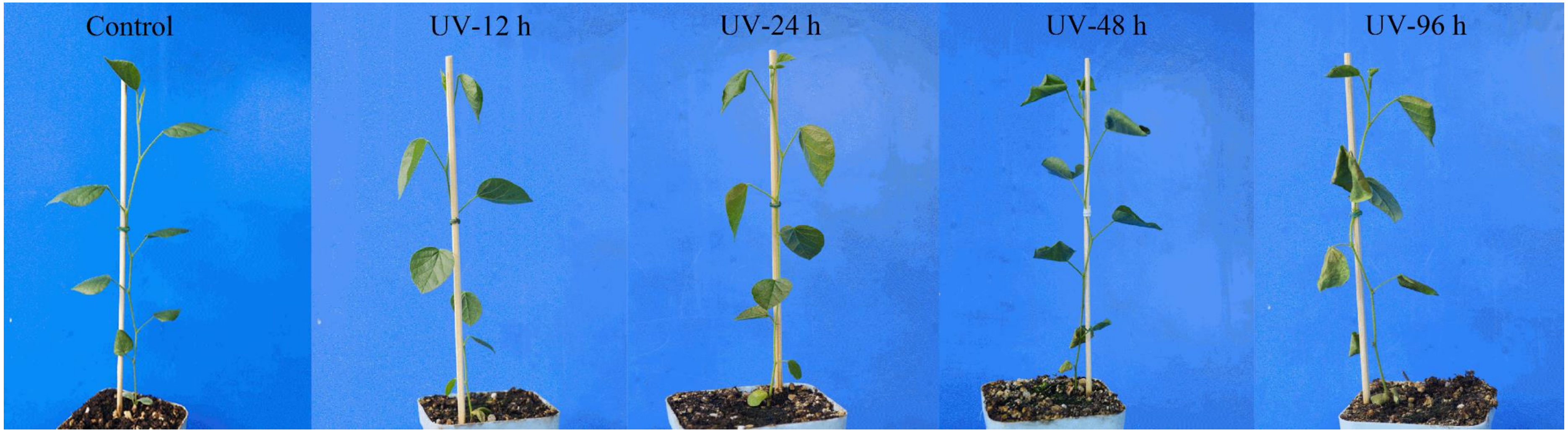

Supplementary Fig. S1. Increasing levels of damage were observed with the elongation of UV-B irradiation time in *G. uralensis* leaves.

Control: healthy plant.

UV-12 h: the top blades curled up.

UV-24 h: the top blades curled up along the midrib, young leaves chlorosis were observed.

UV-48 h: the top blades inactivated, most leaves increasingly curled up, the plants is wilting.

UV-96 h: plant wilted, the area of leaf chlorosis extended, and UV scorch appeared on leaves.

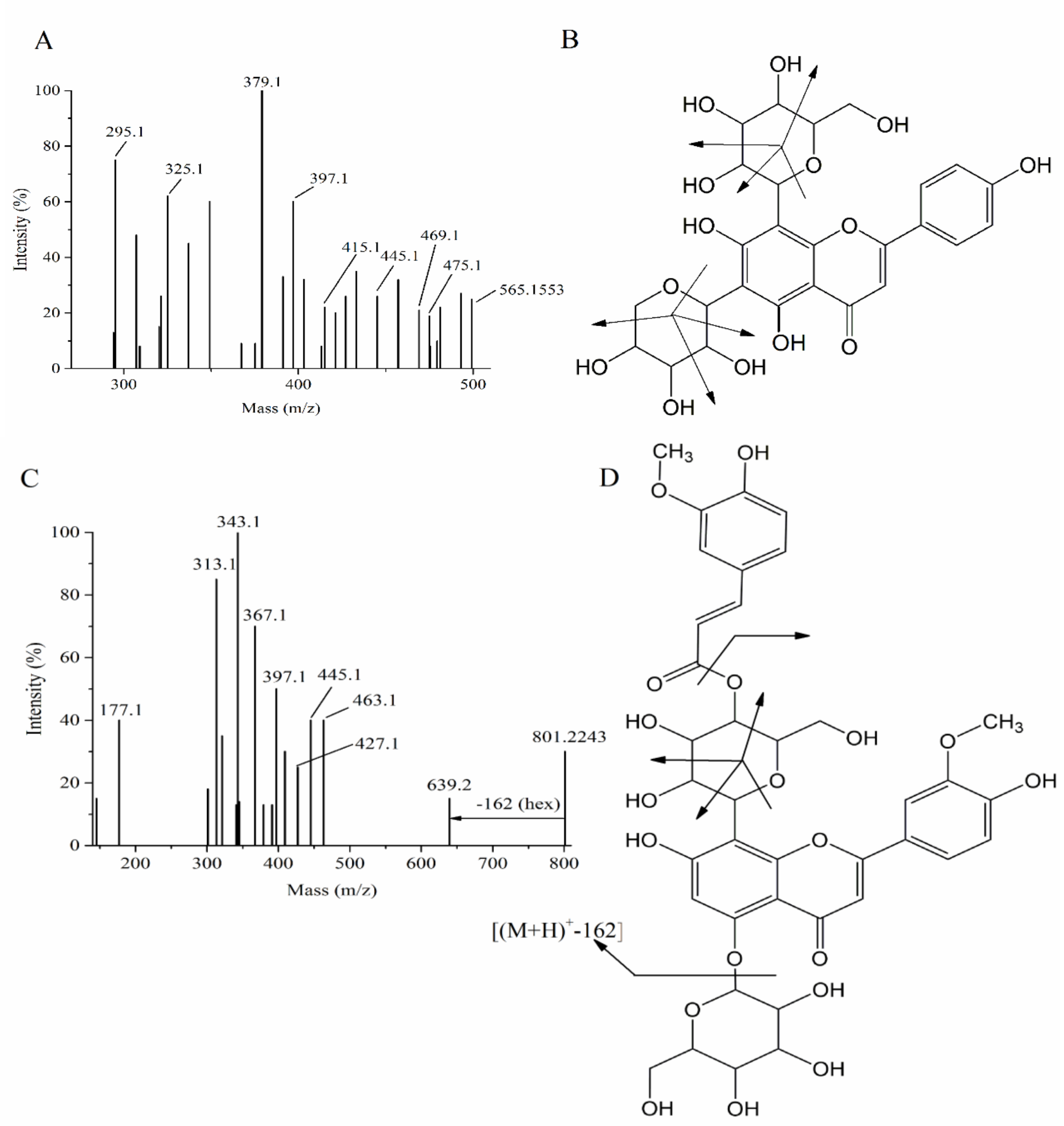

Supplementary Fig. S2. Identification of flavonoids in *Glycyrrhiza uralensis* leaves. (A) MS/MS spectra of *m/z* 565.1553 and the metabolite was putatively identified as *C*-pentosyl-*C*-hexosyl-apigenin. (B) Structure and fragmentation of *C*-pentosyl-*C*-hexosyl-apigenin. (C) MS/MS spectra of *m/z* 801.2243 and the metabolite was identified as *C*-hexosyl-chrysoeriol *O*-feruloylhexoside. (D) Structure and fragmentation of *C*-hexosyl-chrysoeriol *O*-feruloylhexoside.

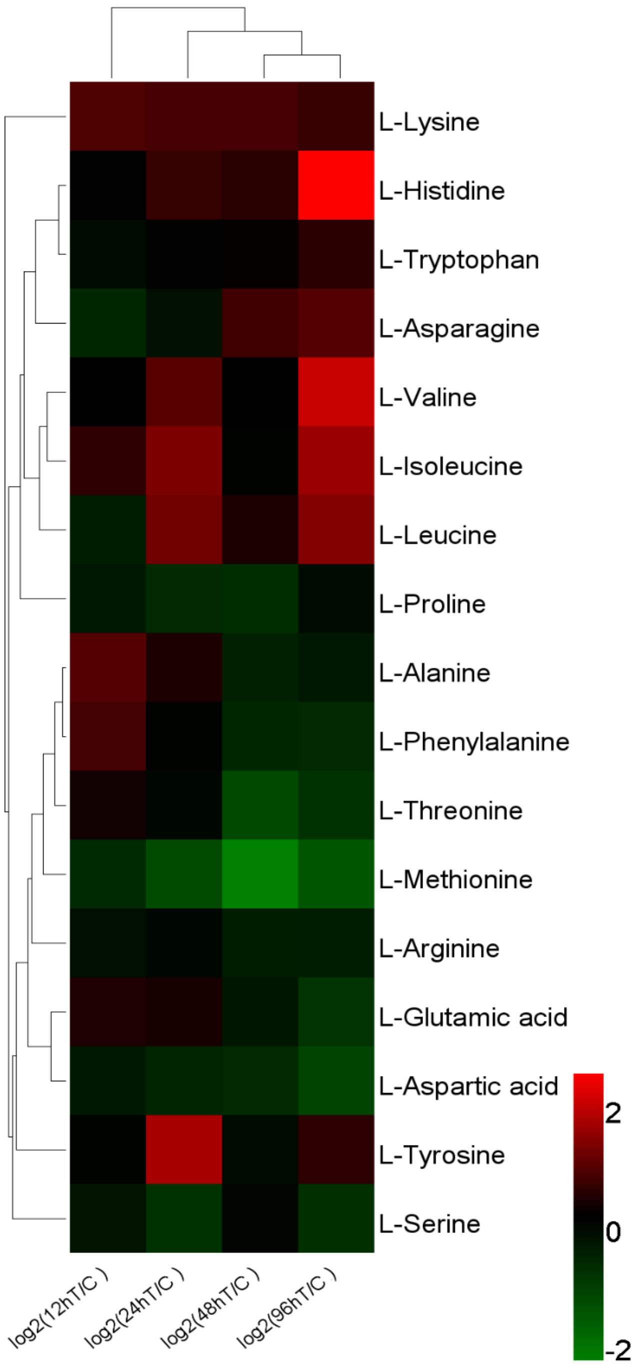

Supplementary Fig. S3. Hierarchical clustering analysis of the fold changes of amino acids in Glycyrrhiza uralensis leaves under UV-B radiation compared to their corresponded controls at four time points. Data of the fold change value of each treatment to control were normalized to complete linkage hierarchical clustering. Each amino acid is represented in a single row, and log_2 (fold change) of each time point is represented in a single row respectively. Red indicates higher contents of this amino acid in treated seedlings than control seedlings, whereas green means relatively lower contents of the amino acid in treated seedlings than control seedlings (color key scale showed on the right of heat map).

Supplementary Table S7. RNA sequencing analysis metrics. Transcriptome analyses were performed in *G. uralensis* leaves treated with UV-B irradiation for 0 (control), 6, 12, 24, 48, and 96 h using an Illumina HiSeq 4000 system.

Sample	Total Nucleotides (bp)	Total Reads (%)	Q20(%)	GC%	adapter(%)	low quality(%)
Control-1	3323286250	26586290 (96.45%)	3236169434 (97.38%)	45.21%	378776 (1.37%)	592966 (2.15%)
Control-2	3251461250	26011690 (96.91%)	3172224386 (97.56%)	45.48%	322874 (1.2%)	498908 (1.86%)
Control-3	3262715750	26101726 (96.66%)	3186172583 (97.65%)	45.77%	421144 (1.56%)	473478 (1.75%)
T6-1	4316737000	34533896 (96.58%)	4226048481 (97.90%)	46.24%	612216 (1.71%)	598406 (1.67%)
T6-2	4127136500	33017092 (96.86%)	4034833031 (97.76%)	46.16%	434306 (1.28%)	623044 (1.83%)
T6-3	3612441750	28899534 (97.01%)	3539187192 (97.97%)	45.52%	434586 (1.45%)	446764 (1.5%)
T12-1	3136418500	25091348 (96.51%)	3070798826 (97.91%)	45.10%	481094 (1.85%)	417940 (1.61%)
T12-2	2758003250	22064026 (97.14%)	2703963935 (98.04%)	44.66%	335590 (1.48%)	306260 (1.35%)
T12-3	3375232750	27001862 (96.73%)	3304676507 (97.91%)	45.17%	466778 (1.67%)	433256 (1.55%)
T24-1	3451002750	27608022 (95.54%)	3389860278 (98.23%)	44.82%	961724 (3.33%)	314044 (1.09%)
T24-2	3022754750	24182038 (97.05%)	2962842462 (98.02%)	45.44%	346866 (1.39%)	375312 (1.51%)
T24-3	3688495250	29507962 (97.08%)	3610940465 (97.90%)	45.24%	381206 (1.25%)	490124 (1.61%)
T48-1	3188344000	25506752 (97.03%)	3120240222 (97.86%)	45.58%	321798 (1.22%)	446802 (1.7%)
T48-2	3211294500	25690356 (96.96%)	3146825417 (97.99%)	45.44%	403762 (1.52%)	388240 (1.47%)
T48-3	3543409000	28347272 (96.72%)	3474803030 (98.06%)	44.90%	527452 (1.8%)	420368 (1.43%)
T96-1	3184614500	25476916 (97.15%)	3122005372 (98.03%)	45.31%	345088 (1.32%)	390680 (1.49%)
T96-2	2696992750	21575942 (96.58%)	2641992640 (97.96%)	45.44%	402374 (1.8%)	350450 (1.57%)
T96-3	3560542250	28484338 (96.4%)	3491976106 (98.07%)	45.48%	652790 (2.21%)	396252 (1.34%)

Supplementary Table S8. List of 10 selected unigenes validated by qPCR. For each, the identities of blasting our RNA-Seq sequences with the *Glycyrrhiza uralensis* genome CDS, and forward and reverse primers used are shown.

Unigene ID (from RNA-Seq)	*G. uralensis* gene ID (from Genome)	Identities of the blasting	Forward / Reverse primer sequence
AK (Unigene0054595)	Glyur003541s00037730	96.8%	CGCGTTGAATCTCTGTGGAG / TGTGGTGGTGGTTGGAAGAA
LKR/SDH (Unigene0065748)	Glyur000012s00000527	99.7%	GTCCAGCCTTCCACCAAGAG / TGCCAAGGATGAGACCACAG
GAD (Unigene0048253)	Glyur000476s00022389	93.6%	AGATGCCGGAGGAGTCGATA / CAGGTTCCATCCATGTGGTC
GSS (Unigene0051101)	Glyur000069s00004063	96.9%	GCACAAGGACAATGCCATCT / CGCCTTCGTCAGAAGATGAT
GPX (Unigene0030909)	Glyur000595s00018295	99.1%	CTTCTTCTCCTTCGCTCATGG / CCACGGTATTGCTCCAGACTC
PAL (Unigene0061071)	Glyur000163s00011136	98.2%	TGCCACACTCAGCAACAAGA / AGGAGCTTGGTGATGGCTTC
C4H (Unigene0023900)	Glyur000002s00000296	99.5%	GCGCAAGATGCGTAGGATAA / CTCGTTGGCCTTGAGGTCTT
CHS (Unigene0052958)	Glyur006062s00044203	98.9%	GCACCGTCACTAGACGCAAG / GGCATGTCCACACCACTTGT
FLS (Unigene0045976)	Glyur002747s00042594	99.6%	AGGCCAGAGACAGAGCAACC / CTTGCCTCCACAATCTCACG
UGT72E2 (Unigene0034774)	Glyur000079s00008902	91.7%	TTCGGTTCGACGACACTCTC / CCAGATCTTGCCACGTGTTC
Guactin			CCTCAACCCAAAGGTCAACAG / GACCAGCGAGATCCAAACGAA
